# Down-regulation of habenular calcium-dependent secretion activator 2 induces despair-like behavior

**DOI:** 10.1038/s41598-021-83310-0

**Published:** 2021-02-12

**Authors:** Hyeijung Yoo, Soo Hyun Yang, Jin Yong Kim, Esther Yang, Hyung Sun Park, Se Jeong Lee, Im Joo Rhyu, Gustavo Turecki, Hyun Woo Lee, Hyun Kim

**Affiliations:** 1 Department of Anatomy, College of Medicine, Korea University, Seoul, 02841 Korea; 2 Department of Biomedical Sciences, Brain Korea 21 FOUR, College of Medicine, Korea University, Seoul, 02841 Korea; 3 Department of Psychiatry, McGill University, Douglas, Mental Health University Institute, Montreal, QC H4H 1R3 Canada

**Keywords:** Neuroscience, Diseases of the nervous system, Depression

## Abstract

Calcium-dependent secretion activator 2 (CAPS2) regulates the trafficking and exocytosis of neuropeptide-containing dense-core vesicles (DCVs). CAPS2 is prominently expressed in the medial habenula (MHb), which is related to depressive behavior; however, how MHb neurons cause depressive symptoms and the role of CAPS2 remains unclear. We hypothesized that dysfunction of MHb CAPS neurons might cause defects in neuropeptide secretion and the activity of monoaminergic centers, resulting in depressive-like behaviors. In this study, we examined (1) CAPS2 expression in the habenula of depression animal models and major depressive disorder patients and (2) the effects of down-regulation of MHb CAPS2 on the animal behaviors, synaptic transmission in the interpeduncular nucleus (IPN), and neuronal activity of monoamine centers. Habenular CAPS2 expression was decreased in the rat chronic restraint stress model, mouse learned helplessness model, and showed tendency to decrease in depression patients who died by suicide. Knockdown of CAPS2 in the mouse habenula evoked despair-like behavior and a reduction of the release of DCVs in the IPN. Neuronal activity of IPN and monoaminergic centers was also reduced. These results implicate MHb CAPS2 as playing a pivotal role in depressive behavior through the regulation of neuropeptide secretion of the MHb-IPN pathway and the activity of monoaminergic centers.

## Introduction

Neuropeptides are essential for a wide range of brain functions and they play critical roles in modulating neuronal activity^1^. For neuropeptides, various calcium-sensing molecules mediate their secretion. In case of the calcium-dependent secretion activator (CAPS; also known as CADPS), it is known to promotes exocytosis of the dense-core vesicles (DCVs) containing neuropeptides with a mechanism involving the tethering of DCVs to the plasma membrane via interaction with phosphatidylinositol 4,5-bisphosphate and SNARE proteins^2–4^.

In vertebrates, there are two types of CAPS (CAPS1 and CAPS2) and they are expressed in a developmental and tissue-specific manner^5,6^. CAPS1 is distributed throughout the central nervous system, but CAPS2 is concentrated in the medial habenula (MHb), cerebellum, cerebrum, and hippocampal areas^5,6^. For the CAPS2 KO mice, there were deficits in the release of brain-derived neurotrophic factor (BDNF) and neurotrophin-3 (NT-3) in the cerebellum, resulting in abnormal cerebellar development and function^7–9^. In the hippocampus, for the CAPS2 KO mice, DCVs accumulated in the presynaptic bouton, leading to impaired adult neurogenesis and development of GABAergic interneuron network^10–12^. The CAPS2 KO mice manifest decreased social interaction with cage mates, a hyperactivity in the home cage, a reduction in exploring, and increased anxiety in novel environments. They also display an aberrant circadian rhythm and exhibit maternal neglect that together indicate autistic-like phenotypes^8^. In depression-related paradigms, CAPS2 KO mice were also more immobile in the forced swim test (FST), indicating increased despair-like symptoms^12^.

The habenula is divided into medial and lateral regions with the MHb being subdivided into dorsal MHb (MHbD) and ventral MHb (MHbV) regions. The MHbD expresses tachykinin 1 (TAC1) gene encoding substance P, neurokinin A; the MHbV expresses choline acetyltransferase (CHAT) gene, encoding choline acetyltransferase. Both MHbD and MHbV expresses TAC2 gene that encodes neurokinin B^13,14^, with the MHbV and MHbD projecting to the medial and lateral parts of the interpeduncular nucleus (IPN), respectively^15,16^. Most of the input to the IPN are from the MHb^15,17^, and glutamatergic and cholinergic inputs from MHb project onto IPN neurons which are predominatly GABAergic^18,19^. The MHb also contains numerous neuropeptides such as tachykinins, VGF, neuropeptide Y, and secretogranin 1–3^20^, and CAPS2 is expressed in both MHbD and MHbV^5,6,21^. In this setting, CAPS2 may play a pivotal role in secretion of MHb neuropeptides.

Many previous studies have reported a relationship between the MHb and depressive behavior with MHbD-lesioned mice showing increased latency to escape, persistence of escape-response after learned helplessness, and displaying anhedonia-like behavior^22,23^. Down-regulation of CHAT in the MHbV also induced depression-like behavior, a decreased sucrose consumption in sucrose preference test (SPT), with no change in FST. By chemogenetic stimulation in this area with designer receptors exclusively activated by designer drugs (DREADD), neuronal activity in the monoaminergic centers was altered, including that of the ventral tegmental area (VTA) and the dorsal raphe nucleus (DRN)^24^.

As MHb is related to neuropsychiatric symptoms^25^, we hypothesized that decreased CAPS2 expression in the MHb might affect neuropeptide signaling and lead to depressive behavior. In the present study, we identified the distribution of CAPS2 in the MHb and its role in depression-related behavioral symptoms. CAPS2 gene was down-regulated in animal models of depression, and selective knockdown of CAPS2 in the MHb induced the despair-like behavior, which may be associated with dysregulation of DCV exocytosis reducing the neuronal activity of the IPN and monoaminergic neurons.

## Results

### Down-regulation of CAPS2 mRNA seen in the habenula of the animal models of depression

**Figure 1:**
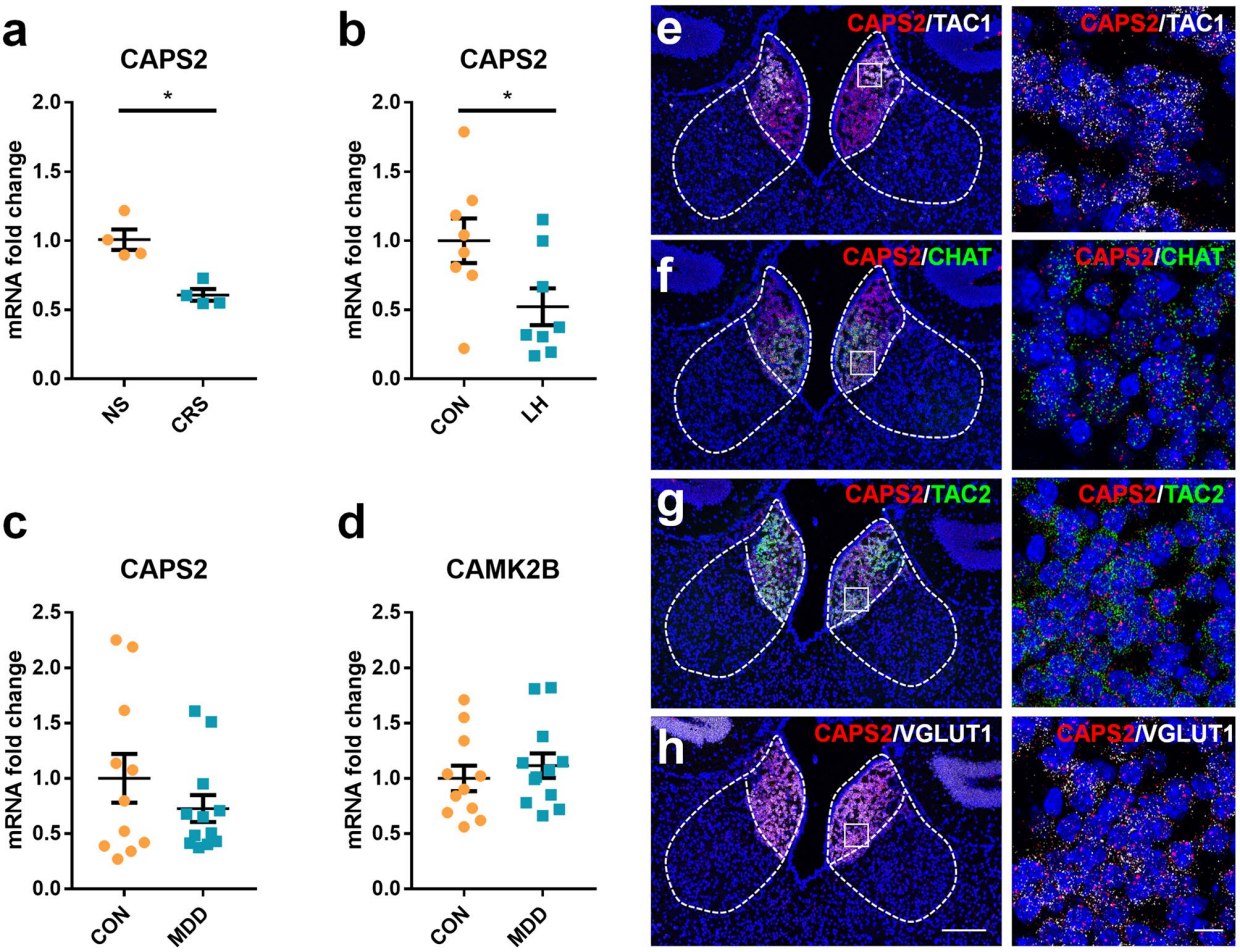
CAPS2 gene expression in the animal models of depression, MDD patients, and identification of CAPS2 expressing cells. (**a–d**) qRT-PCR analysis showed that the CAPS2 mRNA expression level decreased in the habenula of rats exposed to CRS (**a**), mice exposed to electric shock (**b**). CAPS2 mRNA expression showed a tendency to decrease in the habenula of MDD patients who died by suicide (**c** and **d**). CAMK2B levels did not show any difference between the two groups. The relative abundances of mRNA were normalized to the amount of GAPDH using the comparative threshold cycle method. (**e–h**) Photomicrographs of the double-labeled fluorescent in situ hybridization for CAPS2, TAC1, TAC2, CHAT, and VGLUT1. Antisense riboprobe-labeled were for CAPS2/TAC1 (**e**), CAPS2/CHAT (**f**), CAPS2/TAC2 (**g**), and CAPS2/VGLUT1 (**h**). These genes were co-expressed in the MHb. Data represent mean ± SEM, *P < 0.05 (**a**, NS, n = 4; CRS, n = 4 rats; *P* = 0.029, Mann–Whitney *U*-test; **b**, CON, n = 8; LH, n = 8 mice; *P* = 0.038, **c, d**, CON, n = 11; MDD, n = 12 subjects; CAPS2: *P* = 0.279; CAMK2B: *P* = 0.483; Student’s *t*-test; Scale bars, 200 μm; 10 μm in the magnified image).

We previously measured the mRNA levels of the target genes in the habenula of chronic restraint stress (CRS) animal model of depression and postmortem brain samples of MDD patients^24^. We found that CAPS2 mRNA expression was significantly reduced in the habenula of CRS rats compared with those of non-stressed (NS) rats (n = 4 for each group, Mann–Whitney *U*-test; NS vs. CRS expressed as fold change: 1.00 ± 0.14 vs. 0.61 ± 0.09, *P* = 0.029; Fig. 1a) and CAPS2 protein levels in the IPN, a direct downstream of the MHb, also showed a tendency to decrease, being 53.8% of control, as shown via Western blotting (Supplementary Figure S1). CAPS2 mRNA level was also decreased in mice exposed to inescapable electric foot shocks inducing learned helplessness (LH) as compared with control mice (n = 6 for each group, Student’s *t*-test; CON vs. LH expressed as fold change: 1.00 ± 0.46 vs. 0.52 ± 0.38, *T*_(14)_ = 2.285, *P* = 0.038; Fig. 1b). In addition, CAPS2 mRNA levels in postmortem habenula samples of depressive patients tended to decrease, being 73% of non-depressed controls (CON, n = 11; MDD, n = 12; Student’s *t*-test; fold change for CON vs. MDD suicides: for CAPS2, 1.00 ± 0.73 vs. 0.73 ± 0.42, *T*_(21)_ = 1.111, *P* = 0.279; for CAMK2B, 1.00 ± 0.38 vs. 1.11 ± 0.38, *T*_(21)_ = -0.714, *P* = 0.483; Fig. 1c and d).

To characterize the MHb CAPS2-expressing cells, RNAscope assay was performed using the markers of MHb comprising cells (TAC1, CHAT, TAC2) and glutamatergic excitatory neurons (VGLUT1). In this analysis, CAPS2 was expressed in the substance P-ergic neurons in the MHbD (TAC1-positive; 67% of MHbD CAPS2 cells; Fig. 1e) and the cholinergic neurons of the MHbV (CHAT-positive; 90.8% of MHbV CAPS2 cells; Fig. 1f). TAC2, which is expressed throughout the MHb, colocalized with CAPS2 (87.6% of MHb CAPS2 cells, Fig. 1g). CAPS2 signals also colocalized with VGLUT1 in the MHb (89.5% of MHb CAPS2 cells, Fig. 1h). These results suggest that CAPS2-expressing cells are also glutamatergic, cholinergic, and tachykinin-releasing neurons.

### MHb CAPS2 knockdown evokes despair-like behavior

**Figure 2:**
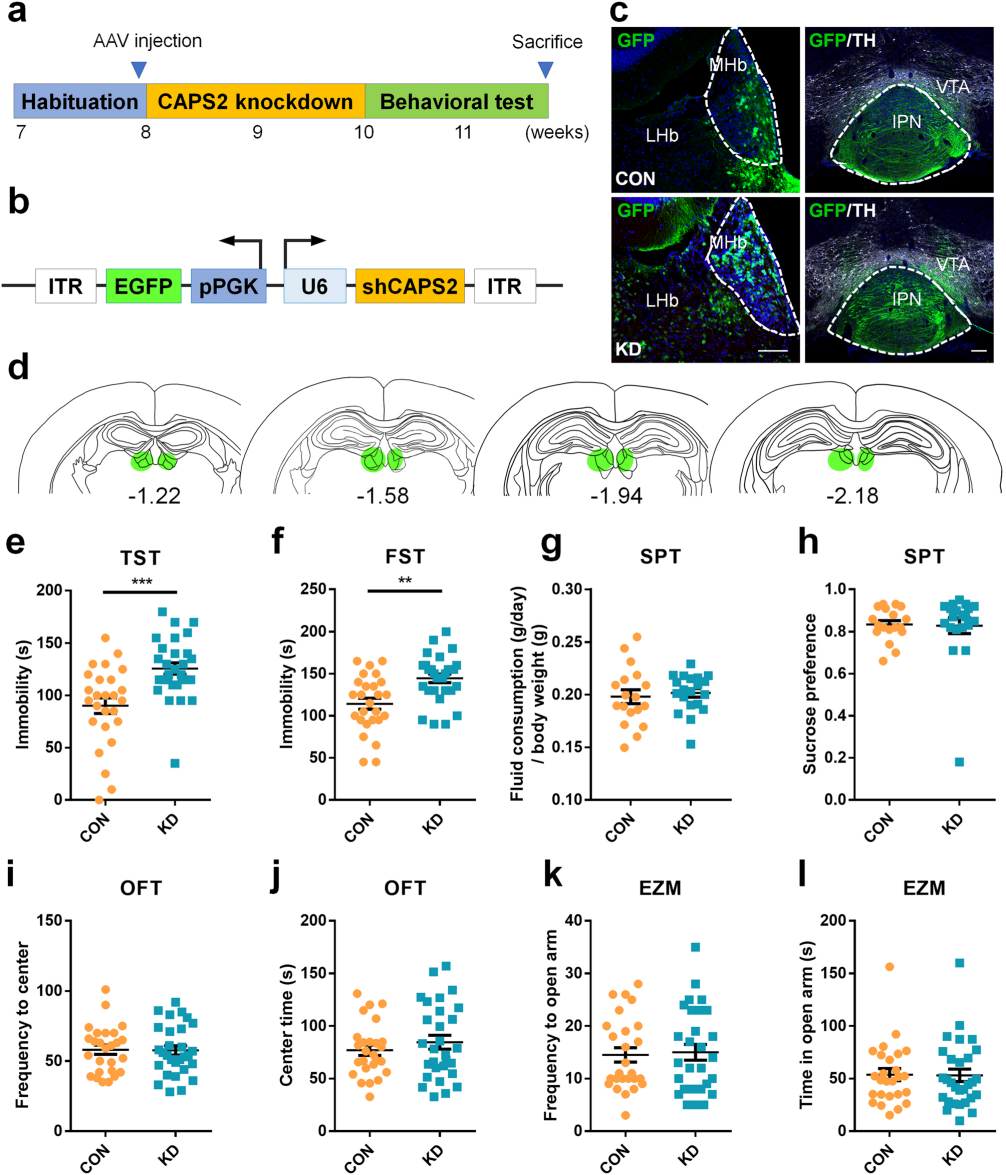
Effects of CAPS2 knockdown in the MHb on anxiety- and depression-like behaviors. (**a**) Experimental paradigm for behavioral testing of mice infected with the virus. (**b**) Schematic representation of the AAV vector engineered to induce CAPS2 knockdown. (**c**) GFP expression of AAV-sh-vehicle (CON) or AAV-sh-CAPS2 (KD) after 4 weeks of injection. Scale bars, 100 μm. (**d**) Diagram showing viral spread in the mouse brain. (**e–l**) Despair-like symptom and anhedonia-like symptom were examined by the tail-suspension test (TST, **e**), forced swim test (FST, **f**), and sucrose preference test (SPT, **g** and **h**). Behavioral effects of expressing AAV-sh-CAPS2 in the MHb on anxiety levels were performed in the open field test (OFT, **i** and **j**) and the elevated zero maze test (EZM, **k** and **l**) and analyzed with Ethovision XT 12. AAV-sh-CAPS2 (KD) injected group showed increased immobility time in TST and FST, indicating despair-like symptoms, but no difference in anxiety-like and anhedonia-like symptom. Data represent mean ± SEM, **P < 0.01, ***P < 0.001 (**e–f**, **i–l**; CON, n = 26; KD, n = 29 mice; TST: *P* < 0.001; FST: *P* = 0.001; OFT, frequency to center: *P* = 0.924, OFT, center time: *P* = 0.379; EZM, frequency to open arm: *P* = 0.807, EZM, time in open arm: *P* = 0.942; **g** and **h**; CON, n = 18; KD, n = 20 mice; SPT, fluid consumption: *P* = 0.637, SPT, sucrose preference: *P* = 0.892; Student’s *t*-test).

We next investigated whether the selective suppression of the MHb CAPS2 expression induced depression-related behaviors. We generated viral vectors to express small interfering RNA (siRNA) targeting the CAPS2 transcript. After testing three CAPS2 knockdown viral vectors in vitro, we selected a sh-CAPS2 that reduced endogenous CAPS2 expression by 50% (Supplementary Table S1). We then injected either adeno-associated virus 2/9 (AAV2/9) containing sh-CAPS2 (KD) or control with empty target sequence (CON) into the mice MHb by stereotaxic injection and checked for injection site (Fig. 2b**–**d). After 2 weeks of injection, behavior tests were done and the expression levels of CAPS2 in the region of interest were assessed (Fig. 2a). Compared with control, the siRNA specifically targeting the CAPS2 transcript effectively reduced CAPS2 protein and mRNA levels (Supplementary Figure S2).

We then evaluated the effects of a MHb-specific CAPS2 knockdown on depression- and anxiety-like behavioral phenotypes. Interestingly, the MHb CAPS2 knockdown resulted in an increased immobility time in both the tail-suspension test (TST) and FST (CON, n = 26; KD, n = 29; Student’s *t*-test; CON vs KD in TST: 90.19 ± 38.45 vs. 125.69 ± 28.96, *T*_(53)_ = -3.891, *P* < 0.001; FST: 114.23 ± 33.64 vs. 144.48 ± 27.59, *T*_(53)_ = -3.661, *P* = 0.001; Fig. 2e and f). However, there was no effect in daily fluid consumption normalized to body weight (CON, n = 18; KD, n = 20; Student’s *t*-test; water plus sucrose solution (g/day) /body weight(g): 0.20 ± 0.028 vs. 0.20 ± 0.018, *T*_(36)_ = -0.475, *P* = 0.637; Fig. 2g) and sucrose preference (CON, n = 18; KD, n = 20; Student’s *t*-test; percentage of the sucrose solution consumed in total fluid intake: 0.83 ± 0.08 vs. 0.83 ± 0.17, *T*_(36)_ = 0.137, *P* = 0.892; Fig. 2h) in SPT, showing no effect on anhedonia-like behavior. Reduced expression of CAPS2 in the MHb did not alter the total distance moved in the open field test (OFT) and elevated zero maze (EZM), implicating no changes in locomotion (Supplementary Figure S3). Time spent in the central area/open arm, the number of center/open arm visits (CON, n = 26; KD, n = 29; Student’s *t*-test; OFT, frequency to center: 58.04 ± 16.94 vs. 57.59 ± 17.96, *T*_(53)_ = 0.096, *P* = 0.924, OFT, center time: 77.06 ± 25.37 vs. 84.52 ± 35.48, *T*_(53)_ = -0.888, *P* = 0.379; EZM, frequency to open arm: 14.50 ± 6.96 vs. 15.00 ± 8.05, *T*_(53)_ = -0.245, *P* = 0.807, EZM, time in open arm: 53.73 ± 29.30 vs. 53.11 ± 31.70, *T*_(53)_ = 0.073, *P* = 0.942; Fig. 2i**–**l), and latency to first visit of a central area/open arm indicating anxiety-like phenotypes were also not changed (Supplementary Figure S3). To further examine the stress-induced anxiety levels, the novelty-suppressed feeding test was performed (Supplementary Figure S4). Latency to bite food pellets and the amount of food consumed after the test did not differ between the groups. As CAPS2 KO mice have manifested autism-like behavior^8^, we next examined the social interaction in the MHb CAPS2 knockdown mice. There was also no significant difference in the social interaction ratio between control and MHb CAPS2 knockdown mice (Supplementary Figure S4). Together, these data suggest that selective reduction of MHb CAPS2 protein induces despair-like behavior, but not anxiety, anhedonia, and social dysfunction.

### MHb CAPS2 knockdown leads to accumulation of presynaptic dense-core vesicles in the IPN

**Figure 3:**
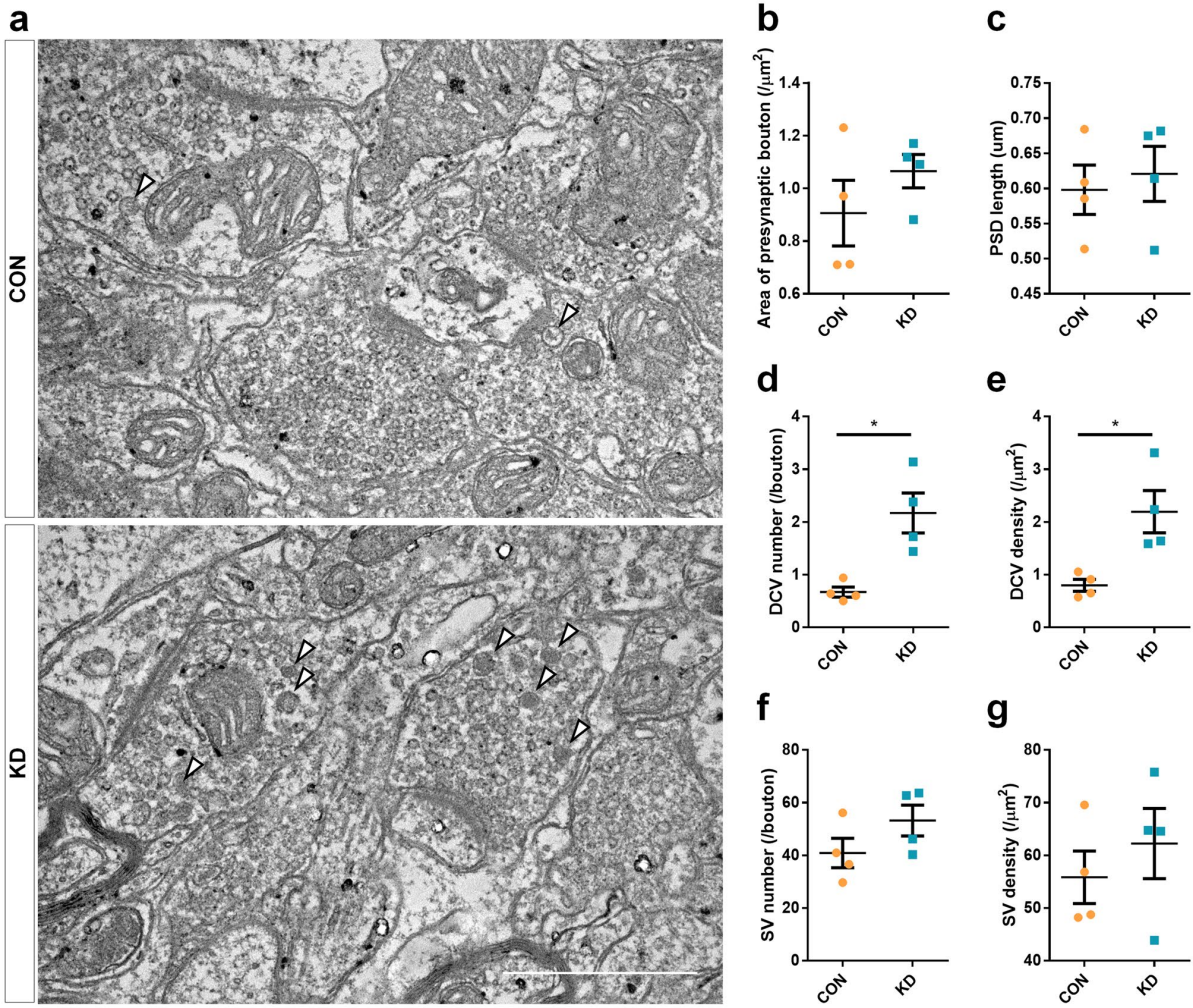
DCVs are accumulated in IPN of MHb CAPS2 knockdown mice. (**a**) Representative electron micrographs of IPN slices from the mice injected with AAV-sh-vehicle (CON) or AAV-sh-CAPS2 (KD). Closed arrowheads indicate DCVs. (**b–g**) MHb CAPS2 knockdown effect in IPN synapses was examined by measuring the area of presynaptic bouton, PSD length, DCV, SV number, and density. Decreased CAPS2 expression in the MHb led to increased DCV number and density in the IPN. Data represent mean ± SEM, *P < 0.05 (CON, n = 4; KD, n = 4; 50 synapses counted and averaged per mice; presynaptic area: *P* = 0.486; PSD length: *P* = 1.000; DCV number: *P* = 0.029; DCV density : *P* = 0.029; SV number: *P* = 0.200; SV density: *P* = 0.686; Mann–Whitney *U*-test; Scale bars, 1 μm).

It is known that the major output of the MHb connects to the IPN through fasciculus retroflexus^15,26^. Since CAPS2 is well-known in regulating DCV exocytosis, we examined the IPN to observe the ultrastructural effect of MHb CAPS2 knockdown using transmission electron microscopy. Areas of presynaptic bouton and length of postsynaptic density did not differ between the two groups (n = 4 for each group; Mann–Whitney *U*-test; area of presynaptic bouton: 0.91 ± 0.25 μm^2^ vs. 1.07 ± 0.13 μm^2^, *P* = 0.486; postsynaptic density length: 0.60 ± 0.07 μm vs. 0.62 ± 0.08 μm, *P* = 1.000; Fig. 3a–c), but DCV number and DCV density both increased in MHb CAPS2 knockdown mice compared with control mice (n = 4 for each group; Mann–Whitney *U*-test; DCV number: 0.67 ± 0.19 vs. 2.17 ± 0.76, *P* = 0.029; DCV density, DCV number per μm^2^ of the presynaptic bouton: 0.80 ± 0.22 vs. 2.19 ± 0.80, *P* = 0.029; Fig. 3a, d, and e). The number of synaptic vesicles (SV) and density of SV showed a slight tendency to increase, but without a significant difference between the two groups (n = 4 for each group; Mann–Whitney *U*-test; SV number: 40.86 ± 11.20 vs. 53.20 ± 11.77, *P* = 0.200; SV density: 55.83 ± 9.96 vs. 62.24 ± 13.31, *P* = 0.686; Fig. 3a, f, and g). This implied that the CAPS2 reduction impairs DCV exocytosis and leads to accumulation of DCVs in the MHb terminals in the IPN.

### MHb CAPS2 knockdown affects c-Fos and p-ERK levels in the IPN

**Figure 4:**
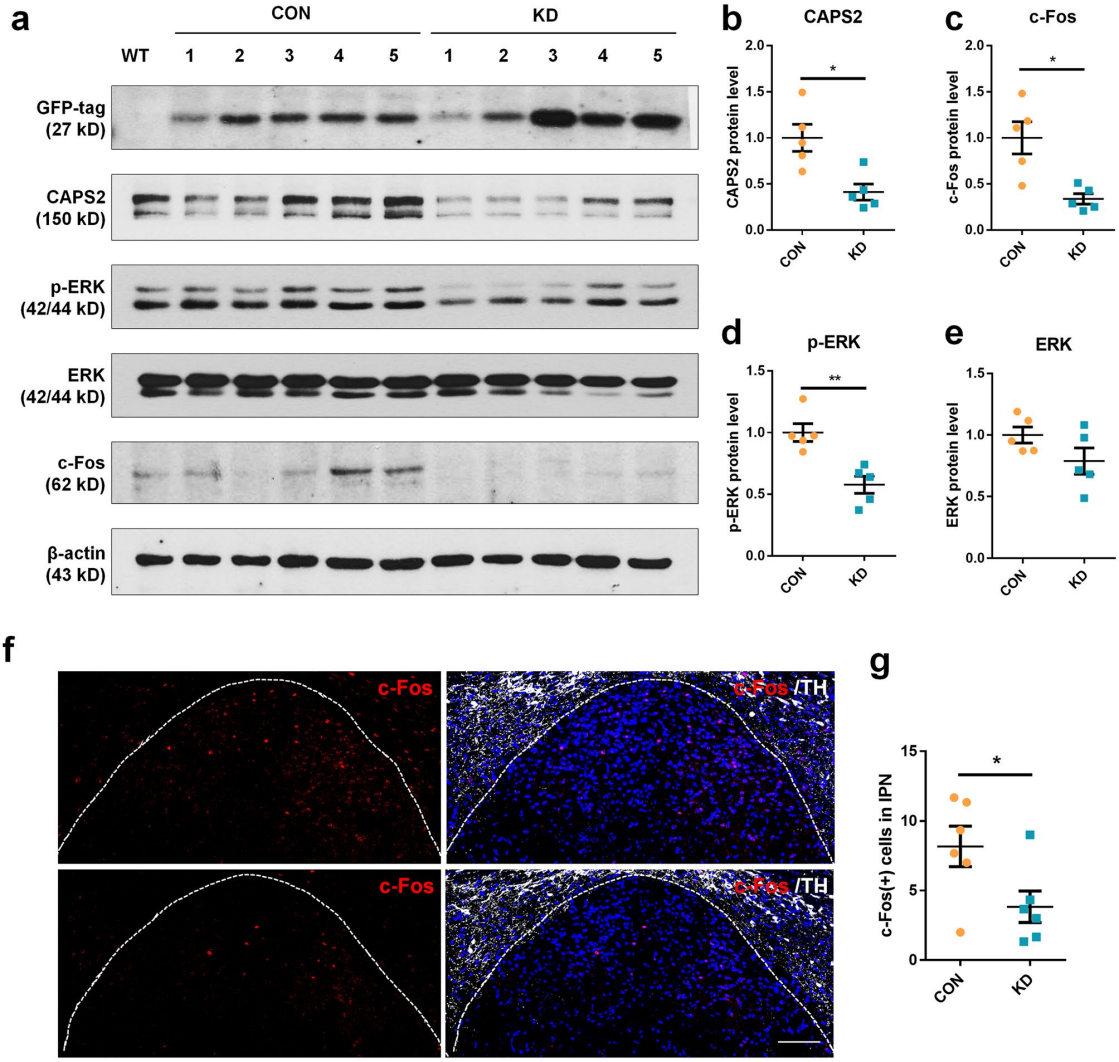
CAPS2 knockdown in the MHb leads to decreased neuronal activity in the IPN. (**a**) Western blot analysis from the IPN tissue of MHb AAV-sh-vehicle (CON) or AAV-sh-CAPS2 (KD) injected mice. (**b**–**e**) Western blot band intensity for CAPS2, c-Fos, p-ERK, ERK. AAV-sh-CAPS2 (KD) virus injection reduced the CAPS2 protein levels in IPN and diminished c-Fos and p-ERK protein levels. Uncropped images are presented in Supplementary Figure S5. (**f** and **g**) Representative immunohistochemistry image (**f**) and c-Fos-positive cell number (**g**) in IPN of control and MHb CAPS2 knockdown mouse. The number of c-Fos-positive cells was decreased in the MHb CAPS2 knockdown group. Data represent mean ± SEM, *P < 0.05, **P < 0.01 (WB, CON, n = 5; KD, n = 5 mice; CAPS2: *P* = 0.016; c-Fos: *P* = 0.016; p-ERK: *P* = 0.008; ERK: *P* = 0.222; Mann–Whitney *U*-test; IHC, CON, n = 6; KD, n = 6; *P* = 0.041, Student’s *t*-test; Scale bars, 100 μm).

Since DCVs accumulate in synaptic bouton of the MHb in the IPN by down-regulation of MHb CAPS2, we measured c-Fos and p-ERK levels as neuronal activity markers in the IPN using Western blot and immunohistochemistry (IHC). In Western blot analysis using IPN lysates containing the presynaptic components derived from the MHb, CAPS2 protein levels were decreased in the knockdown group (n = 5 for each group, Mann–Whitney *U*-test; CAPS2: 1.00 ± 0.33 vs. 0.41 ± 0.20, *P* = 0.016; Fig. 4a and b). Protein levels of c-Fos and p-ERK in the IPN were also decreased, without affecting expression of total ERK (n = 5 for each group, Mann–Whitney *U*-test; c-Fos: 1.00 ± 0.39 vs. 0.34 ± 0.13, *P* = 0.016; p-ERK: 1.00 ± 0.16 vs. 0.57 ± 0.16, *P* = 0.008; ERK: 1.00 ± 0.15 vs. 0.79 ± 0.24, *P* = 0.222; Fig. 4a and c–e). From IHC, the number of c-Fos-positive cells was also decreased in MHb CAPS2 knockdown mice (n = 6 for each group, Student’s *t*-test; c-Fos-positive cells: 8.17 ± 3.56 vs. 3.83 ± 2.78, *T*_(10)_ = -2.351 *P* = 0.041; Fig. 4f and g). These results suggest that presynaptic accumulation of DCV leads to decreased IPN neuronal activity.

### MHb CAPS2 knockdown alters neuronal activity in the monoamine centers

**Figure 5:**
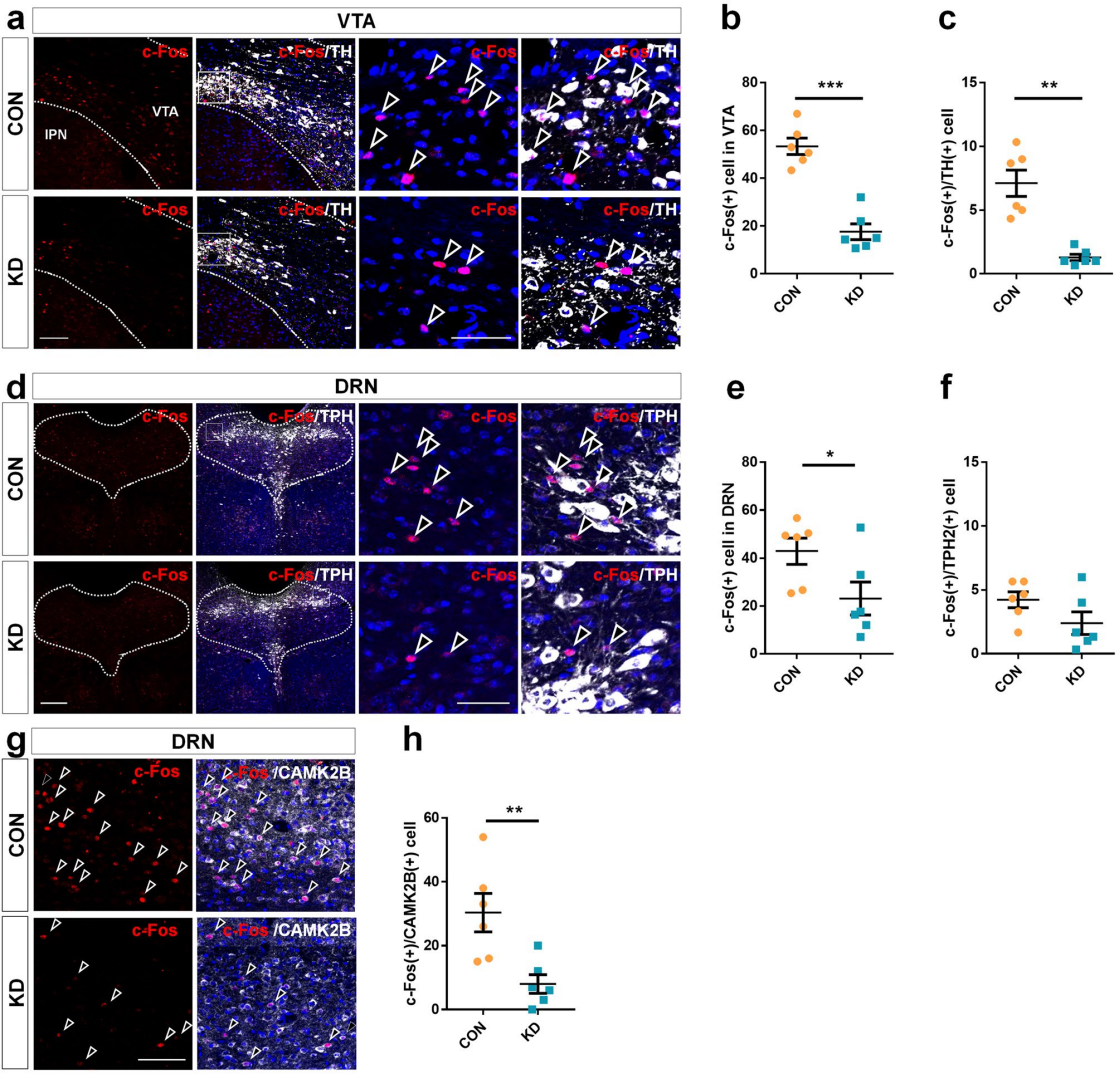
CAPS2 knockdown in the MHb induces altered neuronal activity in the VTA and the DRN. (**a**) Photomicrographs of VTA immunohistochemistry slices from mice injected with AAV-sh-vehicle (CON) or AAV-sh-CAPS2 (KD). MHb CAPS2 knockdown reduced c-Fos positive cells (red) in the VTA neurons. (**b** and **c**) C-Fos-positive cell counts and c-Fos/TH double-positive cell counts in VTA. (**d**) DRN slices from mice injected with AAV-sh-vehicle (CON) or AAV-sh-CAPS2 (KD) were used for immunohistochemistry. (**e** and **f**) c-Fos-positive cell count in DRN was reduced in the knockdown group, but c-Fos/TPH double-positive cell count showed no significant difference. (**g** and **h**) C-Fos/CAMK2B double-positive cells were reduced in DRN of the knockdown mouse. Data represent mean ± SEM, *P < 0.05, **P < 0.01, ***P < 0.001 (CON, n = 6; KD, n = 6 mice; **b**, Student’s *t*-test, *P* < 0.001, **c**, Mann–Whitney *U*-test, *P* = 0.002; Scale bars, 200 μm; 50 μm in the magnified image, 3 sections per mouse is averaged. **e** and **f,** Student’s *t*-test, c-Fos: *P* = 0.049, c-Fos/TPH: *P* = 0.121 each; Scale bars, 200 μm; 50 μm in the magnified image, **h**, Student’s *t*-test, *P* = 0.007; Scale bars, 50 μm).

Monoaminergic centers, including VTA and DRN, are important structures for mood-related behaviors. Recent findings have shown that activating VTA dopamine neurons produce reward, and inhibiting VTA dopamine neurons generate despair-like symptoms^27–29^. VTA receives serotonergic and glutamatergic input from DRN^30,31^, which affects the response to reward^32–34^. As MHb CAPS2 knockdown mice present despair-like symptoms, we thought that neuronal activity in the VTA would differ between the knockdown and control groups. The IHC analysis revealed that indeed c-Fos-positive cell numbers in the VTA were significantly decreased in knockdown mice (n = 6 for each group, Student’s *t*-test; c-Fos-positive cells: 53.33 ± 8.38 vs. 17.61 ± 8.09, *T*_(10)_ = 7.511, *P* < 0.001; Fig. 5a and b). In addition, the number of tyrosine hydroxylase (TH)/c-Fos double-positive cells was significantly decreased in MHb CAPS2 knockdown mice, meaning decreased dopaminergic neuronal activity in the VTA (n = 6 for each group, Mann–Whitney *U*-test; TH/c-Fos double-positive cells: 7.11 ± 2.51 vs. 1.28 ± 0.61, *P* = 0.002; Fig. 5a and c). We also examined neuronal activity changes in the DRN, another major monoaminergic center. The c-Fos-positive cell numbers in the DRN were decreased in the MHb CAPS2 knockdown mice (n = 6 for each group, Student’s *t*-test; c-Fos-positive cells: 42.83 ± 13.35 vs. 23.11 ± 16.91, *T*_(10)_ = 2.242, *P* = 0.049; Fig. 5d and e). However, tryptophan hydroxylase 2 (TPH2)/c-Fos double-positive cells were scant in both groups and did not show any significant differences (n = 6 for each group, Student’s *t*-test; TPH2/c-Fos double-positive cells: 4.22 ± 1.53 vs. 2.39 ± 2.16, *T*_(10)_ = 1.694, *P* = 0.121; Fig. 5d and f). A reduced number of c-Fos positive cells in the DRN is associated with decreased activity in non-serotonergic neurons. The number of c-Fos-positive glutamatergic neurons was found to be significantly lower in MHb CAPS2 knockdown than in controls (n = 6 for each group, Student’s *t*-test; CAMK2B/c-Fos double-positive cells: 30.33 ± 14.73 vs. 8.00 ± 7.12, *T*_(10)_ = 3.342, *P* = 0.007; Fig. 5g and h).

## Discussion

In this study, we demonstrated that a down-regulation of CAPS2 protein in the MHb leads to an increase in the despair-like symptom, one of the core symptoms in the diagnosis of major depressive disorder^35^. CAPS2 expression level is decreased in the CRS rat model, LH mice model of depression, and tended to decline in human MDD, implicating a link for CAPS2 levels in depression. The IPN in MHb CAPS2 knockdown mice manifested an increase in DCV in the presynaptic bouton and decreased neuronal activity. Notably, c-Fos was reduced in monoaminergic centers, including the VTA dopamine neurons and the DRN glutamatergic neurons in MHb CAPS2 knockdown mice.

CAPS2 plays an important role in calcium-dependent synaptic neuropeptide release, and CAPS2 KO mice have shown decreased release of BDNF and NT-3 in the cerebellum, accumulation of DCVs and decreased SVs near active zone in the hippocampus, leading to abnormal synaptic structure and function^7,11,12^. In the MHb, CAPS2-expressing cells co-express TAC1 and TAC2 (Fig. 1e and g), which encode DCV contents such as substance P, neurokinin A, neuropeptide K, and neurokinin B^34^. In addition, a recent peptidogenomic study has reported MHb producing 262 neuropeptides generated from 27 prohormones, such as somatostatin (SST), neuropeptide Y (NPY), and secretogranin 1–3, etc^20^.

The IPN receives the majority of afferents from the MHb^15,17^, and we confirmed the widespread *en passant* projection of MHb AAV-sh-CAPS2 infected axon terminals to the IPN (Fig. 2c). This finding suggests that the accumulation of DCVs at the MHb axon terminals is mediated by CAPS2 deficiency in the MHb (Fig. 3a). Considering that neuropeptides are abundant in the MHb, DCV accumulation at the presynaptic terminals due to the down-regulation of MHb CAPS2 would eventually interfere with the secretion of numerous neuropeptides at the MHb**-**IPN synapses. On the other hand, SV number and density remain unchanged in CAPS2 KD mice, indicating that the MHb CAPS2 knockdown does not affect exocytosis of SV. As evidenced by the decreased level of p-ERK, which is a downstream molecule in neuropeptide signaling^36,37^, MHb CAPS2 knockdown-mediated reduction in neuropeptide signaling led to decreased neuronal activity in the IPN.

Xu et al*.* has reported that the substance P content in rat IPN tissue exposed to chronic mild stress (CMS) increases^38^. This CMS-mediated increase of substance P in IPN tissues may be a result of accumulation due to impaired DCV exocytosis caused by CAPS2 reduction. In the same study, the firing rate of IPN neurons in response to substance P perfusion was recorded, and the spontaneous activity after substance P perfusion was increased by approximately 41% of the baseline^38^. Since TAC1, a gene producing substance P, is enriched in MHbD and the activity of IPN neurons is augmented by substance P, the reduction in c-Fos expression in IPN neurons by MHb CAPS2 knockdown (Fig. 4) may be partially related to the impaired exocytic release of substance P, which is consistent with the results of Xu et al.. Although their roles in the habenula are not known for other neuropeptides, they have been reported to be involved in mood changes such as depression. For example, SST is significantly decreased in CSF of depressed patients^39,40^, and SST expression is reduced in the anterior cingulate cortex of MDD patients^41^. Also, the disinhibition of SST positive interneurons leads to reduced anxiety in the elevated plus maze and despair-like behavior in FST^42^. In addition, intracerebroventricular injection of NPY and NPY1R agonist reduced despair-like symptoms in FST^43,44^.

The CAPS2 gene has also been reported to be associated with neuropsychiatric symptoms. Aberrant CAPS2 splicing, leading to lack of the complete sequence of exon 3, has been detected in a subgroup of autistic patients. CAPS2 KO mice also manifested autistic phenotypes, including impaired social interaction, decreased exploratory behavior and increased anxiety in a novel environment, plus increased immobility time in FST^8,12,45^. Nonetheless, the reduction in CAPS2 expression in the habenula did not show any defects in social interaction (Supplementary Figure S4), and habenula lesions, including both MHb and LHb, also did not affect social interaction^46^. We assume that the despair-like symptom in CAPS2 KO mice is partially due to the lack of habenular CAPS2 function, and the reduction of calcium-dependent docking of DCVs to the plasma membrane in the MHb**-**IPN synapses might have prevented neuropeptide signaling and led to decreased neuronal activity in the IPN and the behavioral change.

It is realized that monoaminergic centers, including VTA and DRN, are important structures in mood-related behaviors. Inhibition of VTA dopamine neurons using optogenetic tools induce increased despair-like symptom and stimulation of VTA dopamine neurons rescues despair-like symptom in the mouse CMS model^29,47^. In recent studies, the release of dual serotonin and glutamate by optical activation from DRN serotonergic neurons projecting to the VTA led to the release of dopamine in the nucleus accumbens and established conditioned place preference^30^. In addition, optogenetic activation of DRN non-serotonergic glutamatergic neurons elicits reward-related behavior, suggesting that it is glutamatergic transmission that makes up the majority of the DRN-VTA pathway producing reward-seeking behavior^31,48^.

Although there was no significant activity change in DRN serotonergic neurons in MHb CAPS2 knockdown mice (c-Fos expression of TPH2-positive neurons, Fig. 5d and f), it is notable that glutamatergic neuronal activity was significantly reduced by MHb CAPS2 knockdown (Fig. 5g and h). Recently, the afferent connections of the DRN, conveyed by the IPN, were systemically investigated with sensitive tracers, and demonstrated that the GABAergic neurons of the IPN input into DRN GAD67 expressing neurons^15^. Since GABAergic interneurons of DRN form an internal circuit and modulate DRN serotonergic neurons^49–51^, it is reasonable to assume that DRN glutamatergic neurons are also regulated by DRN GABAergic interneurons. One plausible possibility is that the reduced activity of IPN GABAergic neurons by MHb CAPS2 knockdown may increase the activity of DRN GABAergic neurons and lead to disinhibition of DRN glutamatergic neurons. Furthermore, since IPN mainly forms connections with GABAergic neurons in various brain regions such as the median raphe, nucleus incertus, supramammillary nucleus, septum, and laterodorsal tegmental nucleus^15^, IPN GABAergic neurons could be indirectly regulating the neuronal activity of the DRN and the VTA through various pathways.

Many studies have reported that MHb is closely linked to various neuropsychiatric symptoms^25,52^. We have also previously reported that reduced cholinergic signaling by MHb CHAT knockdown leads to anhedonia but not despair-like behavior^24^. Also, genetic ablation of a key transcription factor leading to the lesion of MHbD, which is abundant in substance P-expressing neurons, resulted in anhedonia-like behavior^22^. It is interesting to note that MHb CAPS2 knockdown induces only despair-like behavior as opposed to that of CHAT knockdown. The results of the above studies suggest that the MHb is a remarkably complex structure, and the functions of dorsal and ventral parts of the MHb act independently or in coordination in affecting emotional behavior. The details of the close relationship between neuronal signaling in the MHbD and MHbV in the MHb-IPN pathway are currently not known. The IPN also has a complex structure compared to its size with an efferent pathway to various brain regions. As such, further research on defining the effects of various neuropeptides and neurotransmitters on synaptic plasticity concerning the IPN neurons and their impact on emotion-related behaviors is warranted.

In this study, we showed that reduced neuropeptide signaling in the MHb-IPN circuit induces despair-like symptoms and changes in neuronal activity in monoaminergic centers. These findings bring to focus the mechanistic importance of the MHb and originating neuropeptide secretion on depression circuitry and functional pathways.

## Methods

### Animals

All experimental procedures with animals were approved by the Korea University Institution of Animal Care and Use Committee (Study approval number KOREA-2018–0079-C1) and were performed according to the guidelines of Korea University and the ARRIVE (Animal Research: Reporting of In Vivo Experiments) guidelines. Adult male C57BL/6 mice and adult Sprague–Dawley male rats (Japan SLC, Inc., Shizuoka, Japan) were housed one per cage under a 12-h light/dark cycle (lights on at 8 a.m.) and given ad libitum access to food and water.

### Animal model of depression and human subject samples

Animals were randomly divided into experimental and control groups. We used cDNA library generated from mRNA of rats exposed to chronic restrain stress and human patients with MDD^24^. LH model was generated as previously reported^53^. Detailed methods for developing rat CRS and mice LH depression animal models are described in supplementary information. Post-mortem habenular tissue was obtained by the Douglas-Bell Canada Brain Bank (www.douglasbrainbank.ca; Douglas Institute, McGill University, Canada) following banking guidelines of the *Fonds de Recherche du Québec Santé* and with approval of the Douglas Institute Ethics Research Board. Brains were collected after obtaining informed consent from next-of-kin. For demographic characterization of human subjects, refer to our prior study^24^.

### qRT-PCR

qRT-PCR was done as previously described^24^. The habenula was isolated from the animal brain immediately after decapitation and placed in TRIzol solution (Ambion, Austin, TX, USA). Total RNA sample (2 μg) was reverse-transcribed into cDNA using Moloney Murine Leukemia Virus reverse-transcriptase (M-MLV RT; Promega, Madison, WI, USA) and oligo (dT) primer (Novagen, Milwaukee, WI, USA). qRT-PCR was performed with 0.5 μg of the RT product in presence of specific primer sets (Supplementary Table S2). PCR amplification with iQ SYBR Green Supermix was performed in triplicate using the CFX96 Touch-Time System (Bio-Rad, Hercules, CA, USA). Final products of qPCR were electrophoresed on 2% agarose gels and visualized with SafeView Nucleic Acid Stain (G108, Applied Biological Materials, Canada). The cycle numbers (C_t_) of the critical point at which the fluorescent signal exceeded the background were determined by qRT-PCR, and expression values for each gene were normalized to expression values of GAPDH, the endogenous control within each sample. Relative quantification used to calculate the fold change was performed using the comparative C_t_ method (ΔΔC_t_).

### Viral vector and administration into mouse brain

The AAV-sh-vehicle and AAV-sh-CAPS2 were constructed using pAAV-U6-GFP vector (provided by Cell Biolabs, Inc., San Diego, CA, USA), containing a U6 promoter expressing shRNA and a PGK promoter expressing EGFP. The sequences of CAPS2 shRNA were 5′-GCTCCATTACAGCTTTGCATT-3′ (Supplementary Table S1, U6-sh-CAPS2-1), 5′-CCCAGATTCATCTCGAAAGAA-3′ (U6-sh-CAPS2-2), and 5′-GCGCTGCAAATGTTCGTCTTT-3′ (U6-sh-CAPS2-3). Detailed methods for the cell culture and in vitro knockdown experiments are in supplementary material. For viral injection, eight weeks-old mice were anesthetized with 1% isoflurane and placed in a stereotaxic apparatus (Ultra-precise stereotaxic instruments for mice; Stoelting Co., Wood Dale, IL, USA). Mice were injected bilaterally with 1 μl of 10^12^ to 10^13^/ ul concentrated AAV viral solution into the MHb (coordinates from bregma: − 1.34 mm anterior/posterior, ± 0.83 mm medial/lateral, − 3.05 mm dorsal/ventral, with 10° angle toward the midline in the coronal plane) using microinjection cannula (30 gauge, Plastics One, Roanoke, VA, USA) and UltraMicroPump III (World Precision Instruments, Sarasota, FL, USA) at 100–120 nl/min. Behavior experiments were performed at least 14 days after surgery. The injection sites were examined at the end of the behavior tests, and only data from animals with correct injections were included.

### Behavioral paradigms

Depression behavior studies were done as previously described^24,53^.

#### Sucrose preference test

Single-housed mice were habituated with two identical water bottles for a day and then were exposed to two bottles for three days, one with 1% sucrose and the other with tap water. Sucrose and water consumption were recorded daily by re-weighing the two bottles. Sucrose preference was calculated as a relative ratio of mass of sucrose solution intake/total fluid intake.

#### Open field test

Mice were habituated for at least 30 min to the testing room and placed in the corner of the open field arena (45 cm × 45 cm × 40 cm). Total distance moved, time spent, frequency visiting, latency to the center arena was measured using Ethovision XT 12 tracking software (Noldus, Wageningen, Netherland).

#### Elevated zero maze test

Mice were placed in the closed quadrant of the maze and were allowed to explore the maze for 5 min. Total distance travelled, time spent, frequency visiting, latency to open quadrant were analyzed by Ethovision XT 12.

#### Tail-suspension test

Tail-suspension test was conducted in a 4-chamber apparatus divided by acrylic partitions, and the mice were suspended in each chamber by the tail. A video was recorded for 6 min, and the last 4 min were scored for immobility time.

#### Forced swim test

Mice were exposed to a clear cylinder filled with 24–25 °C water for 6 min and immobility time was scored for the last 4 min. The cylinder was 45 cm in diameter and 60 cm high.

#### Social interaction test

One day before the test, mice were allowed to explore the open arena, in which two cages were placed for acclimatization to the novelty of the environment. On the test day, a cage with a social object (a mouse) was placed on one side of the arena and another cage with a non-social object (marbles) on the other side. The level of sociality was assessed by measuring the frequency and the time mice engaged in interaction with each cage.

#### Novelty suppressed feeding test

Each mouse was weighed and deprived of food for 24 h. In the plastic cage (30 cm × 50 cm × 20 cm) with standard bedding, the food pellet is placed on a white filter paper. The latency for the mouse to start eating the pellet under bright pin-point light is recorded. After the test, the mice were moved to their home cages but pre-weighed to measure the food amount eaten.

### RNAscope assay

Frozen brain sections (14 μm thick) were cut coronally. Sections were then thaw-mounted onto Superfrost Plus Microscope Slides (Fisher Scientific, Waltham, MA, USA). The slides were post-fixed in 4% paraformaldehyde (PFA) and dehydrated in ascending concentration of ethanol, then treated with protease. For RNA detection, RNAscope fluorescent Multiplex detection reagents (ACDBio, Newark, CA, US) were used as in previous studies and following the manufacture’s recommendations^54^. Detailed methods and probes used for RNAscope are described in the supplementary information.

### Electron microscopy

The animal was anesthetized with alfaxalone and xylazine and transcardially perfused with 0.9% NaCl before 2% PFA, and 2.5% glutaraldehyde in 0.1 M phosphate buffer (pH 7.4). The brain was removed and post-fixed overnight at 4 °C. The IPN was dissected from the fixed brain and washed with the same buffer two times, and treated with 1% osmium tetroxide for 90 min. The tissues were dehydrated with an ethanol series in ascending concentration, propylene oxide, and embedded in epon mixture (Oken Shoji, Tokyo, Japan). Polymerized blocks were trimmed, and the region of interest was selected. Thin sections (70 nm) were made using Leica EM UC6 ultramicrotome (Leica Microsystems), mounted on 200 mesh copper grids, stained with 2% uranyl acetate and 1% lead citrate for 5 min each. For each sample, 50 presynaptic boutons with post-synaptic density were randomly selected and observed under a transmission electron microscope (Hitachi H-7650; Hitachi, Tokyo, Japan) at the accelerating voltage of 80 kV.

### Immunohistochemistry

Mice were anesthetized and perfused transcardially with heparinized 0.9% NaCl, then with 4% PFA in phosphate-buffered saline (PBS). Mouse brain was harvested and post-fixed in 4% PFA, then in 30% sucrose in PBS solution at 4 °C. After being cryosectioned to 40 μm thick sections, it was blocked for 1 h in 0.2% Triton X-100, 3% bovine serum albumin in PBS. The sections were incubated with primary antibody overnight at 4℃, and the secondary antibody was applied for 1 h at room temperature. After mounting the slices, the sections were observed on Zeiss LSM700 (Zeiss, Oberkochen, Germany) confocal microscope.

### Antibodies

The antibodies for experiments were as follows; CAPS2 (ab69794), TPH2 (ab111828), CAMK2B (ab52476), GFP (ab290; ab13970) (all from Abcam, Cambridge, UK), c-Fos (for WB, NBP2-50,037; Novus biologicals, Briarwood, CO, USA; for IHC, sc-52-G; Santa Cruz Biotechnology, Dallas, TX, USA), phospho-ERK1/2 (p-ERK, Thr202/Tyr204, #9101), ERK1/2 (#9102; Cell Signaling Technology, Danvers, MA, USA), β-actin (SC-47787; Santa Cruz Biotechnology), TH (AB152; EMD Millipore, Temecula, CA, USA). Secondary antibodies used include donkey anti-goat cy3 (Jackson, 1:500), donkey anti-rabbit 647 (Jackson, 1:500), and donkey anti-chick 488 (Invitrogen, 1:500).

### Western blot

Habenula and IPN tissues were obtained from a cryo-section containing the habenula region using a punch. The tissues were lysed with sodium dodecyl sulfate (SDS) lysis buffer containing 4% SDS, 125 mM Tris–HCl (pH 6.8), 20% glycerol, and 0.004% bromophenol blue. For the immunoblotting, 7.5 μg of proteins were used. Membrane was cut before antibody hybridization.

### Statistics

Statistical analysis was conducted with IBM SPSS Statistics 24 for Windows software (IBM co., Armonk, NY, USA). Comparison between two groups were analyzed with the two-tailed unpaired Student’s *t*-test. The Mann–Whitney *U*-test was used for datasets with sample size ≤ 5, datasets that does not satisfy a normal distribution or equality of variance by Shapiro–Wilk test or Levene’s test, respectively. *P* < 0.05 was considered significant, and values were expressed as mean ± SEM.

## Supplementary Information

Supplementary Information.

## Data Availability

The data that support the findings of this study are available from the corresponding author upon reasonable request.
