# Supplementary material for: Down-regulation of habenular calcium-dependent secretion activator 2 induces despair-like behavior
Source: Sci Rep. 2021 Feb 12;11:3700. doi: 10.1038/s41598-021-83310-0 (PMC7881199; doi:10.1038/s41598-021-83310-0)
Supplement: Supplementary file 1 — Supplementary Information. [file 41598_2021_83310_MOESM1_ESM.docx]

**SUPPLEMENTARY INFORMATION**

**Title:** Down-regulation of habenular calcium-dependent secretion activator 2 induces despair-like behavior

**Authors and affiliation:**

Hyeijung Yoo^1^**^†^**, Soo Hyun Yang^1^**^†^**, Jin Yong Kim^1^, Esther Yang^1^, Hyung Sun Park^1^, Se Jeong Lee^1^, Im Joo Rhyu^1,2^, Gustavo Turecki^3^, Hyun Woo Lee^1,2^*, and Hyun Kim^1,2^*

^1^Department of Anatomy, College of Medicine, Korea University, Seoul 02841, Korea. ^2^Department of Biomedical Sciences, Brain Korea 21 PLUS, College of Medicine, Korea University, Seoul 02841, Korea.

^3^Department of Psychiatry, McGill University, Douglas, Mental Health University Institute, Montreal, QC H4H 1R3, Canada

^†^These authors contributed equally to this work.

***Corresponding authors**

Hyun Woo Lee, Ph.D.

Tel: 82-2-2286-1385

Fax: 82-2-929-5696

E-mail: biocais@korea.ac.kr

Hyun Kim, MD. Ph.D.

Tel: 82-2-2286-1153

Fax: 82-2-929-5696

E-mail: kimhyun@korea.ac.kr

**Supplementary Figures and Tables**


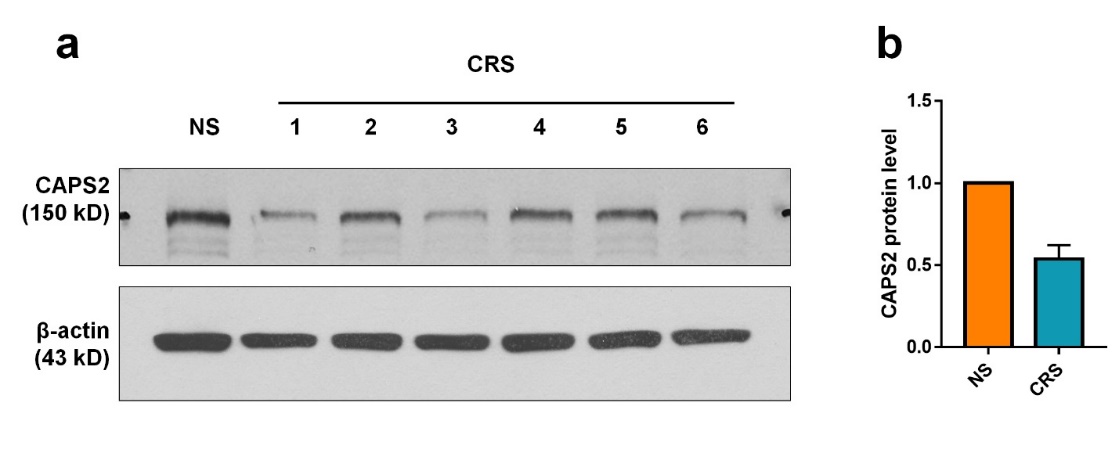


**Supplementary Figure S1. CAPS2 protein level decreased in rats exposed to chronic restraint stress.**

(**a**) Western blot from rat IPN samples of animals exposed to chronic restraint stress for consecutive 14 days. (**b**) CRS reduced the CAPS2 protein level to 53.8% of control (NS). Data represent mean ± SEM (NS, n = 6, non-stressed rat IPN samples that were pooled; CRS, n = 6 rats).

| **Plasmid vector** | **Fold change**  **(qPCR 1^st^)** | **Fold change**  **(qPCR 2^nd^)** |
| --- | --- | --- |
| U6-GFP | 1 | 1 |
| U6-CAPS2-① | 0.4892 | 0.5779 |
| U6-CAPS2-② | 0.8647 | 0.7629 |
| U6-CAPS2-③ | 0.6628 | 0.6397 |

**Supplementary Table S1. *in vitro* validation of CAPS2 knockdown.**

qRT-PCR analysis of AAV vectors engineered to induce CAPS2 knockdown. Three candidates for CAPS2 knockdown vectors were transfected in Neuro2A cell line, and endogenous CAPS2 expression was suppressed. The most efficient vector U6-CAPS2-① was selected and used to produce the AAV virus.


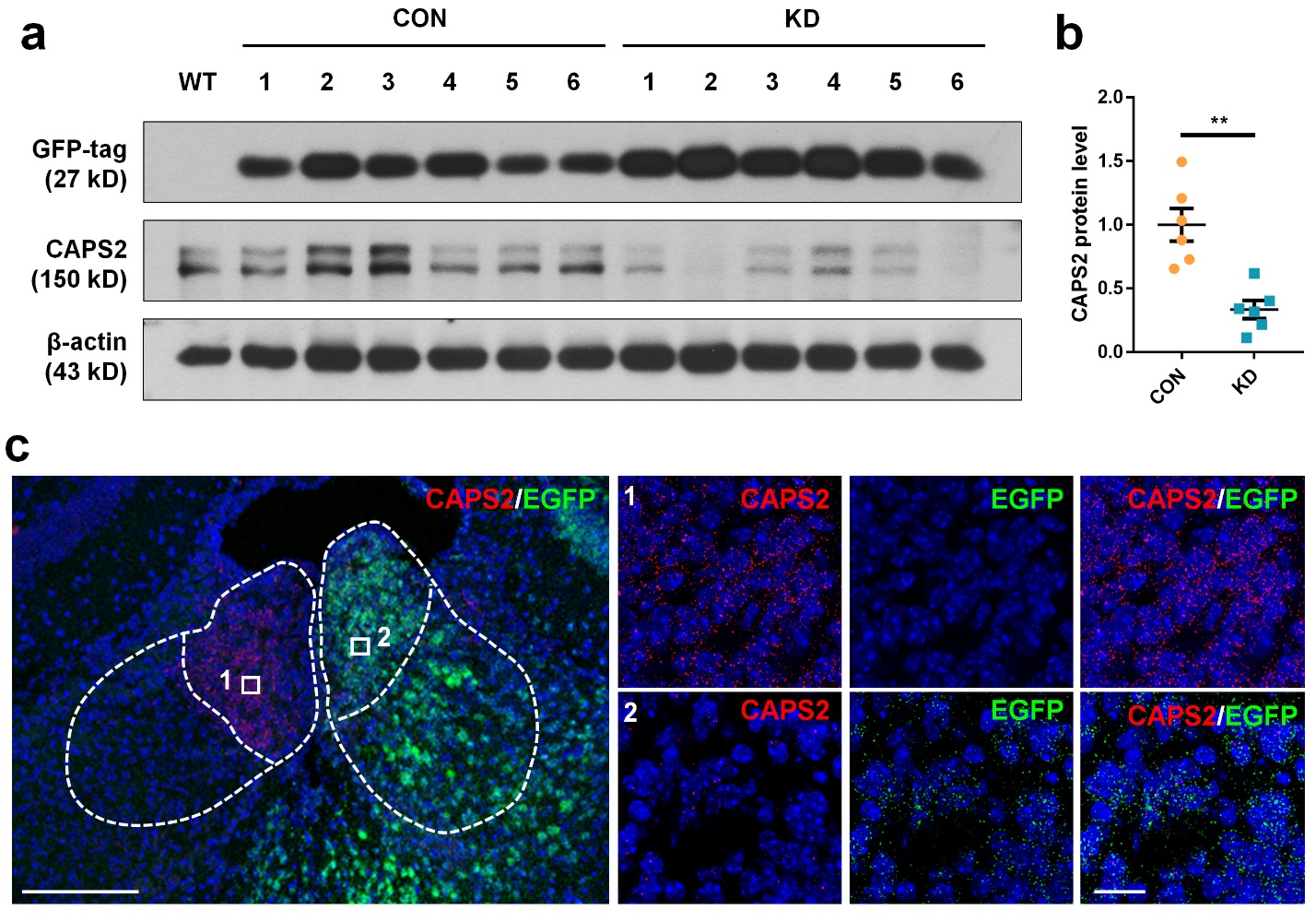


**Supplementary Figure S2. *in vivo* validation of CAPS2 knockdown efficiency.**

(**a** and **b**) Western blot analysis of mice habenula injected with AAV-sh-vehicle (CON) or AAV-sh-CAPS2 (KD). CAPS2 band intensity of knockdown group was reduced to 37% of control. Data represent mean ± SEM (CON, n = 6, KD, n = 6 mice, Student’s *t*-test; *T*_(10)_ = 4.542, *P* = 0.001). (**c**) RNAscope was performed on mouse brain slices from mice ipsilaterally injected with AAV-sh-CAPS2 (KD). CAPS2 (red) and GFP (green) mRNA were visualized using counterstaining with Hoechst (blue). 1: virus non-injected site, 2: KD virus injected site. Scale bars, left panel: 200 μm; right panel: 10 μm.


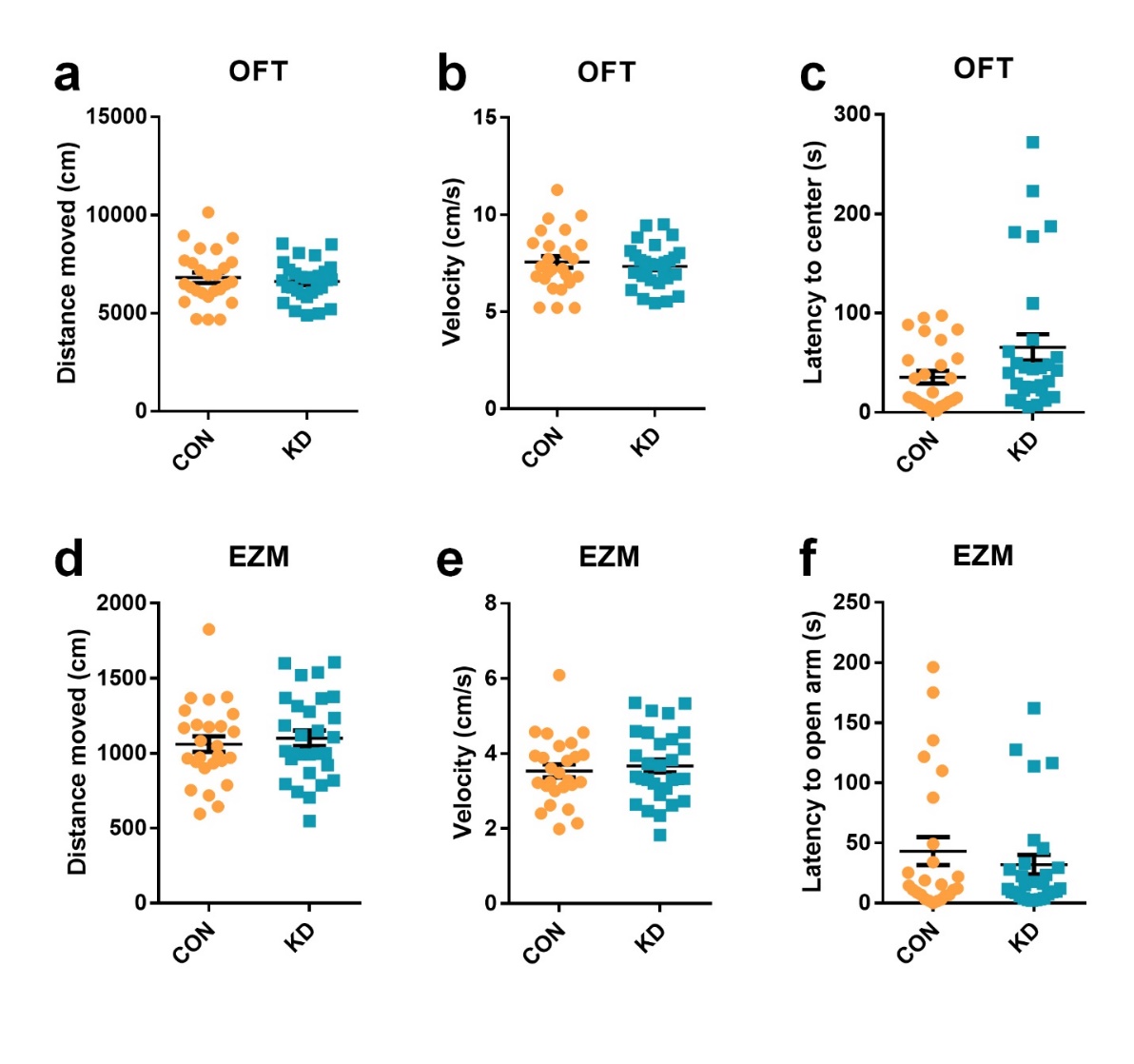


**Supplementary Figure S3. Effects of MHb CAPS2 knockdown on locomotion and anxiety-like behaviors.**

(**a**−**c**) In the open field test, CAPS2 knockdown mice showed no difference in distance moved, frequency to the central area, and latency to the central area, compared with controls. (**d**−**f**) In the elevated zero maze, CAPS2 knockdown mice showed no change in distance moved, frequency to open arm, and latency to open arm, compared to controls. Data was measured using Ethovision XT12. Data represent mean ± SEM (CON, n = 29; KD, n = 26 mice)

**
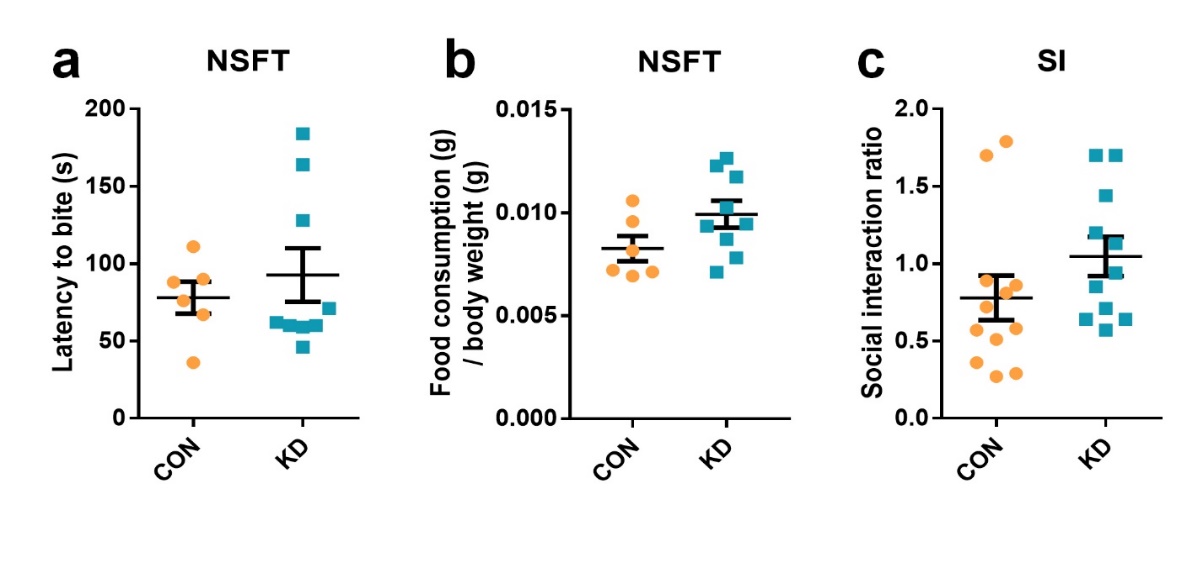
**

**Supplementary Figure S4. Effects of MHb CAPS2 knockdown on the stress-mediated anxiety-like behavior and sociality.**

(**a** and **b**) In the novelty suppressed feeding test, CAPS2 knockdown mice showed no difference in latency to bite and amount of food consumption normalized to weight. (**c**) CAPS2 knockdown mice did not show any change in the social interaction (SI) ratio with the novel mouse. Social interaction time was measured by Ethovision XT 12. Data represent mean ± SEM (**a**, **b**, CON, n = 6; KD, n = 9 mice; **c**, CON, n = 12; KD, n = 11 mice).


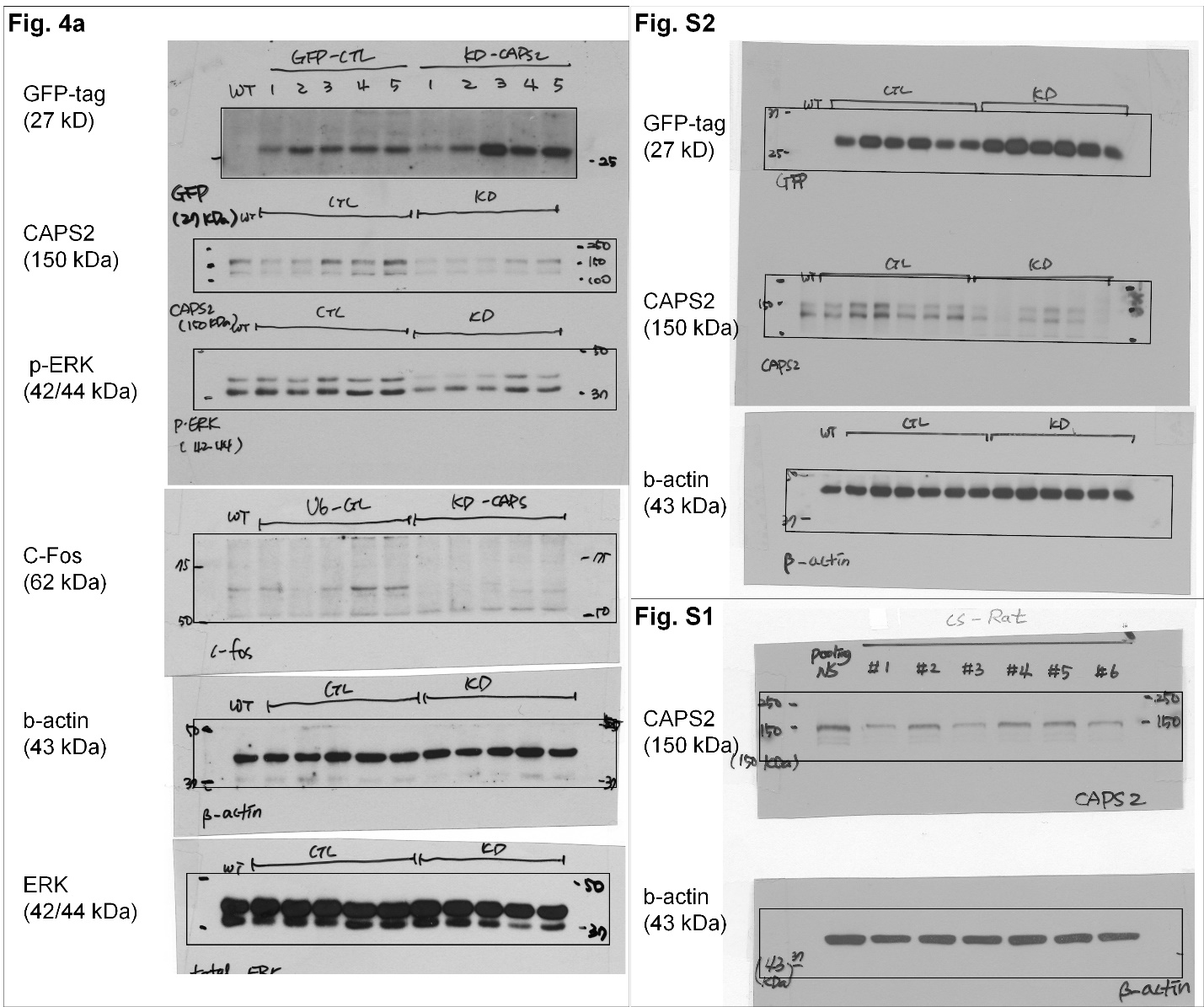


**Supplementary Figure S5. Uncropped images for Fig. 4a., S1, S2 Western blot.**

| **Name** | **Primer** | **Sequence** | **Size** |
| --- | --- | --- | --- |
| GAPDH  (Rat) | FW | 5’- AGT TCA ACG GCA CAG TCA AG -3’ | 118bp |
|  | RV | 5’- TAC TCA GCA CCA GCA TCA CC -3’ |  |
| CAPS2  (Rat) | FW | 5’- AAA CAA CTG ACT TGC GCA TCC -3’ | 147bp |
|  | RV | 5’- CGC AAT GAC TTC TTT CAC GGT -3’ |  |
| GAPDH  (Mouse) | FW | 5’-ACC CAG AAG ACT GTG GAT GG-3’ | 171bp |
|  | RV | 5’-CAC ATT GGG GGT AGG AAC AC-3’ |  |
| CAPS2  (Mouse) | FW | 5'-TAC TGC TTT CCC TTC GGA CG-3' | 98bp |
|  | RV | 5'-TCT GCG GGC CCT TCT ATT TT-3' |  |
| GADPH  (Human) | FW | 5’-ACC CAC TCC TCC ACC TTT GAC-3’’ | 110bp |
|  | RV | 5’- TCC ACC ACC CTG TTG CTG TAG -3’ |  |
| CAPS2  (Human) | FW | 5’-CAC TAG AGG CTC AAC CGC AA-3’ | 147bp |
|  | RV | 5’-CAT AGC GGA CAA CCA AGG GT-3’ |  |
| CAMK2  (Human) | FW | 5’-AAG AAC AGC AAG CCG ATC CA-3’ | 91bp |
|  | RV | 5’-TGA GCC GGA TGT AAG CGA TG-3’ |  |

**Supplementary Table S2. Quantitative RT-PCR primer set**

Primer sets used in Quantitative RT-PCR for CRS rat, LH mouse depression model, and MDD patients.

**Supplementary methods**

**Chronic restraint stress model**

The procedure was performed according to the chronic restraint stress induction protocol as described previously. Animals were randomly assigned to either of experimental group of control group. An experimental group (chronic restraint stress, CRS) were exposed to restraint stress for two hours per day for two weeks. A control group (non-stressed, NS) remained in their home cage and handled for 5 min daily. A breathable decapicone (DC 200, DecapiCones, Braintree Scientific, Braintree, MA, USA) was used for restraint.

**Learned helplessness model**

Mice were exposed to foot shock sessions for 3 days. During the foot shock session, 100 inescapable electric foot shocks were delivered at an intensity of 0.3 mA for 5 seconds and an inter-shock interval of 5–99 s in shock chambers(chamber dimensions, 30 cm × 30 cm × 25 cm; TSE, Bad Homburg, Germany). Control mice were placed in the shock chamber for the same time without the foot shock.

**Cell culture and in vitro knock down experiment for validation of CAPS2**

Mouse brain neuroblast Neuro2A cells were purchased from American Type Culture Collection (Manassas, VA). Neuro2A cells were cultured in Eagle’s Minimum Essential Medium containing 10% fetal bovine serum (Invitrogen, Carlsbad, CA, USA) and maintained at 37°C in a humidified incubator containing 95% air and 5% CO_2_. For validation of CAPS2 knockdown plasmid, Neuro2A cell lines were transfected with AAV-U6-sh-vehicle, AAV-U6-sh-CAPS2-①, AAV-U6-sh-CAPS2-② and AAV-U6-sh-CAPS2-③. Plasmids were transfected into cells by using lipofectamine 2000 (Invitrogen) according to the manufacturer’s instructions. Neuro-2a cells were transiently transfected with EGFP-expressing plasmids containing shRNA. After incubation for 3 days, EGFP-positive cells were checked with a fluorescence microscope, and then qPCR was performed, and to compare the mRNA expression of endogenous CAPS2 to identify the plasmid with the highest knockdown efficiency.

**RNAscope assay and analysis**

Mice were anesthetized and brains are dissected and frozen using isopentane on dry ice. Frozen brain sections (14 μm thick) were cut coronally. Sections were then thaw-mounted onto Superfrost Plus Microscope Slides (Fisher Scientific, Waltham, MA, USA). The slides were post-fixed in 4% paraformaldehyde (PFA) for 10 min and dehydrated in ascending concentration of ethanol for 5 min, then air-dried. The sections were then treated with protease for 10 min. For RNA detection, RNAscope fluorescent Multiplex detection reagents (ACDBio, Newark, CA, USA) were used following the manufacture’s recommendations. Probe hybridization and amplification were performed using the HybEZ hybridization oven (Advanced Cell Diagnostics, Hayward, CA, USA). The labeled probes were conjugated to Alexa Fluor 488, Atto 550, and Atto 647. The sections were hybridized with the labeled probe mixture at 40°C for 2 hours per slide. Unbound hybridization probes were removed by washing the sections three times with 1x wash buffer at room temperature for 2 min. Following steps for signal amplification included incubations at 40°C with Amplifier 1-FL for 30 min, with Amplifier 2-FL for 15 min, with Amplifier 3-FL for 30 min, and with Amplifier 4 Alt B-FL for 15 min. Each amplifier solution was removed by washing with 1x wash buffer at room temperature for 2 min. The slides were viewed, analyzed, and photographed with TCS SP8 Dichroic/CS (Leica Microsystems, Wetzlar, Germany). The sequences for probe generation were as follows: CAPS2, 285−1462 of NM_001252109.1 (NCBI Gene accession number); TAC1, 20−1034 of NM_009311.2; TAC2, 15−684 of NM_009312.2; CHAT, 1090−1952 of NM_009891.2; VGLUT1, 464−1415 of NM_182993.2; and EGFP, 628−1352 of U55763.1 (Advanced Cell Diagnostics). RNAscope images were quantified using HALO image analysis software, FISH v2.1.8 (Indica Labs.). HALO’s FISH module can quantify probe signals and co-expression of up to four fluorescent probes on a per cell, including cell classification (0, 1+, 2+, 3+ and 4+) based on ACD Bio’s recommended RNAscope scoring guidelines. After designating region of interest (ROI), colocalization was determined by dual labeling of CAPS2 and gene of interest in the same DAPI signal indicating individual cells.
